# Investigating causal relationships between Body Mass Index and risk of atopic dermatitis: a Mendelian randomization analysis

**DOI:** 10.1038/s41598-020-72301-2

**Published:** 2020-09-17

**Authors:** Yik Weng Yew, Marie Loh, Steven Tien Guan Thng, John C. Chambers

**Affiliations:** 1 National Skin Centre, 1 Mandalay Road, Singapore, 308205 Singapore; 2 Lee Kong Chian School of Medicine, NTU, Singapore, Singapore; 3 Imperial College London, London, UK; 4 Skin Research Institute Singapore, Singapore, Singapore

**Keywords:** Obesity, Epidemiology

## Abstract

Population studies suggest that atopic dermatitis (AD) is associated with an increased risk of obesity, however a causal relationship between these two conditions remains to be established. We therefore use Mendelian randomization (MR) to evaluate whether obesity and AD are causally interlinked. We used summary statistics extracted from genome wide association studies of Body Mass Index (BMI) and AD. MR analysis was performed in both directions to establish the direction of causality between BMI and AD. We find that genetically determined increase in adiposity is associated with increased risk of AD (odds ratio of AD 1.08 [95% CI 1.01 to 1.14; p = 0.015] per unit increase in BMI). Conversely, genetically determined increased risk of AD is not associated with a higher BMI (change in BMI attributable to AD based on genetic information: 0.00; 95% CI − 0.02 to 0.02; p = 0.862). There was no evidence for confounding of these genetic analyses by horizontal pleiotropy. Our results indicate that the association of AD with obesity is likely to reflect a causal role for adiposity in the development of AD. Our findings enhance understanding of the etiology of AD, and the basis for experimental studies to evaluate the mechanistic pathways by which adiposity promotes AD.

## Introduction

Atopic dermatitis (AD) is a common chronic inflammatory skin disease with significant patient and population burden. It currently affects 20% of children and 10% of adults in the developed world^1^. It is characterised by itch and skin inflammation^2^ reflecting the underlying epidermal barrier dysfunction^3,4^ and immune dysregulation of the skin^5^. Increasingly, it has been reported to be a systemic disease and observed to be associated with other chronic co-morbidities that have immunological basis, such as asthma and allergic rhinitis, as well as metabolic and psychological disturbances^6^.

AD has been reported to be associated with the presence of obesity in many observational epidemiological cohort studies. In a recent meta-analysis, it has been reported that patients who were obese had close to 1.5 times higher odds of having AD^7^. The rising global prevalence of AD, also closely parallels increasing global burden of obesity^8,9^. However, these predominantly cross sectional and observational studies may be limited by confounding factors such as adverse demographic or environmental exposures, and also cannot exclude reverse causation^10,11^. Whether BMI has a causal role in the development of AD therefore remains uncertain.

Causality between an exposure and outcome can be evaluated or estimated with a study design known as Mendelian randomization (MR)^12,13^. This approach investigates causal relationships by using inherited genetic variants as instrumental variables that influence exposure status. As these genetic variants are randomly allocated at point of conception, they are analogous to the randomization process in controlled trials and are less affected by problems of confounding factors and reverse causation. In this study, we therefore used genetic associations and concept of Mendelian randomization (MR) to evaluate the causal relationships between AD and obesity, as measured by Body Mass Index (BMI, a widely used measure of adiposity).

## Results

**Figure 1:**
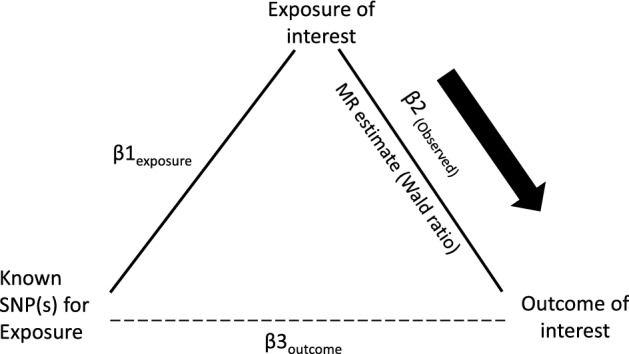
Schematic diagram of Mendelian randomization (MR) experiment of exposure upon outcome of interest. Known genetic instruments (SNPs) for exposure were used as instrumental variables to assess the causal effect of exposure upon outcomes. Β1_exposure_ is the estimated coefficient from the regression of exposure on the genetic variant(s) using the exposure GWAS. Β2_(Observed)_ is the observed coefficient of the relationship between the exposure and outcome of interest. Β3_outcome_ is the regression coefficient of outcome on the corresponding genetic variant using the outcome GWAS. MR estimate is the wald ratio of Β3_outcome_/Β1_exposure_. Significant (*P* < 1 × 10^−8^) and near-independent BMI SNPs (based on an approximate conditional and joint multiple-SNP (COJO) analysis that takes into account LD (linkage disequilibrium) between SNPs at a given locus) from BMI GWAS were used as instrumental variables to assess the causal effect of BMI upon AD while significant (*P* < 5 × 10^−8^) and independent AD SNPs (at least 4 MB(mega base pairs) apart) from AD GWAS were used as instrumental variables to assess the causal effect of AD upon BMI.

Our experimental design is summarized in Fig. 1. In brief, we carried out bi-directional MR analysis using genetic data from two recently published genome-wide association study (GWAS) datasets of BMI and AD in accordance to recent proposed guidelines for reporting MR analysis^14–17^.

### Effect of BMI upon AD

We identified genetic variants influencing BMI (BMI SNPs) for use as genetic instruments to evaluate the effect of adiposity on risk of AD in our MR experiment. (Supplementary Fig. S1a). We used the most recent published GWAS for BMI which comprises the meta-analysis between data from the GIANT consortium and the UK Biobank study (N = 681,275 participants). There were a total of 941 SNPs that were associated with BMI at P < 1 × 10^–8^(Supplementary Table S1). For the genetic effects on BMI, we used the results of the conditional and joint multiple SNP (COJO) analysis. We then evaluated the relationship between these BMI SNPs and AD using effect estimates from a recent GWAS of AD (N = 21,000 cases and 95,000 controls). Results for MR analyses are summarized in Table 1. The MR estimate of BMI on AD using inverse variance weighted (IVW) analysis yielded an odds ratio of 1.08 (95% CI 1.01 to 1.14; p = 0.015) for having AD per unit increase in BMI (kg/m^2^). The maximum-likelihood MR estimate gave a similar odds ratio for AD of 1.08 (95% CI 1.01 to 1.14; p = 0.015) per unit increase of BMI (kg/m^2^). The weighted median based method and MR-Egger regression analysis yielded odd ratios of 1.07 (95% CI 0.97 to 1.18) and 1.06 (95% CI 0.96 to 1.16) respectively. However, neither reached statistical significance (p = 0.195 and p = 0.248 respectively). The MR-Egger regression analysis gave no significant evidence of horizontal pleiotropy, with the intercept being not significantly deviated from zero (intercept: 0.000; 95% CI − 0.002 to 0.003; p = 0.663).

**Table 1:** Summary results of Mendelian randomization (MR) analysis using various methods.

MR method	Parameter	Beta or OR (95% CI)	P value
**Risk of atopic dermatitis (AD) per unit (kg/m**^**2**^**) increase in BMI Based on MR**			
IVW (random effects)	OR	1.08 (1.01 to 1.14)	0.015
IVW (random effects, modified*)	OR	1.12 (1.04 to 1.21)	0.004
Maximum likelihood method	OR	1.08 (1.01 to 1.14)	0.015
Weighted median method	OR	1.07 (0.97 to 1.18)	0.195
MR Egger (random effect)	OR	1.06 (0.96 to 1.16)	0.248
**Unit (kg/m**^**2**^**) change in BMI among those who had AD compared to controls**			
IVW (random effects)	β	0.00 (− 0.02 to 0.02)	0.862
IVW (random effects, modified*)	β	0.00 (− 0.02 to 0.02)	0.982
Maximum likelihood method	β	0.00 (− 0.03 to 0.02)	0.868
Weighted median method	β	− 0.01 (− 0.03 to 0.01)	0.312
MR Egger (random effect)	β	0.04 (− 0.02 to 0.11)	0.189

*Excluding possible pleiotropic genetic factors in analysis.

As part of sensitivity analyses, we repeated the MR studies removing SNPs with pleiotropic effects that might confound the relationship between BMI and AD (see “Methods” section). This modified IVW analysis yielded a similar odds ratio for AD per unit increase in BMI (kg/m^2^) of 1.12 (95% CI 1.04 to 1.21). As SNPs identified by the COJO analysis could potentially include non-independent variants in high linkage disequilibrium (LD), as a further sensitivity analysis, we systematically excluded SNPs across a range of r^2^ thresholds (r^2^ > 0.1, 0.2 and 0.5) followed by distance pruning at < 500 kb as well as SNPs with r^2^ thresholds (r^2^ > 0.001 and > 0.01) within 1000 kb apart (Supplementary Table S2a). MR estimates obtained were statistically significant and consistent with results obtained when all 941 GWAS BMI SNPs were analysed (Supplementary Table S2b).

### Effect of AD upon BMI

We next identified genetic variants influencing AD (AD SNPs) for use as genetic instruments to assess whether AD has a causal effect on BMI. We identified 24 AD SNPs from the results of the EAGLE consortium dataset. (Supplementary Fig. S1b, Supplementary Table S3) MR results are summarized in Table 1. The MR estimate for the effect of AD on BMI using the IVW method yielded an effect estimate of 0.00 unit change in BMI (kg/m^2^) among those who had AD compared to controls (95% CI − 0.02 to 0.02; p = 0.862). The maximum-likelihood MR estimate gave a similar effect estimate of 0.00 (95% CI − 0.03 to 0.02; p = 0.868). The weighted median based method and MR-Egger regression analysis yielded effect estimates of − 0.01 (95% CI − 0.03 to 0.01) and 0.04 (95% CI − 0.02 to 0.11) respectively. None reached statistical significance (p = 0.312 and p = 0.189 respectively). The MR-Egger regression analysis gave no significant evidence of horizontal pleiotropy, as the intercept was not significantly deviated from zero (Intercept: − 0.00; 95% CI − 0.01 to 0.00; p = 0.152). Modified IVW analysis after excluding potential horizontal pleiotropic SNPs, based upon published associations (see “Methods” section), yielded a similar effect estimate of 0.00 (95% CI − 0.02 to 0.02).

### No measurement error [NOME] assumption

To assess how measurement errors in SNP exposure estimates might affect our results, we estimated the attenuation of effect estimates in IVW and MR Egger MR methods using *F* statistics and I^2^_GX_ respectively (see “Methods” section). Both statistics range from 0 to 100%, with values close to 100% suggestive of minimal attenuation.

For the IVW analyses, we find almost no attenuation of effect estimates (*F*_GX_ − 1/*F*_GX−_ = 99.6% and 93.1% for the BMI to AD and AD to BMI MR analyses respectively). The I^2^_GX_ estimate for MR Egger analysis (BMI to AD) was 93.6%, suggesting that any measurement errors in the SNP BMI associations did not attenuate the effect estimates to a large degree. In contrast, the I^2^_GX_ estimate for AD to BMI was 4%, suggesting that MR estimate derived from MR-Egger may be attenuated by measurement errors in AD, and is therefore less robust in evaluating MR estimates.

## Discussion

Using Mendelian randomization, we showed that a higher BMI was causally associated with an increased risk of AD. In contrast, there was no evidence for a causal relationship of AD with an increase in BMI. We demonstrated that these findings are based upon valid primary MR assumptions and were also robust across different MR methods^12,18,19^.

Our results are consistent with the findings of prospective observational birth/infant cohort studies^20,21^. BMI at infancy or fat mass at birth is associated with an increased risk of a subsequent AD diagnosis, with other prospective observational studies of children also reporting that presence of obesity was associated with subsequent development of AD^22–25^. Although cross-sectional studies in adults support a relationship between AD and obesity, the direction of causality is uncertain^26^. Mendelian randomization thus provides a unique opportunity to examine this directionality.

Our mendelian randomization study suggested that BMI may have a causal role in the development of AD. The underlying mechanisms for this phenomenon could be secondary to the pro-inflammatory state and impaired epidermal barrier status of obesity^27–29^. The body adipose tissue contributes to the persistent low-grade inflammation by production of inflammatory cytokines^27,28,30,31^. Adipokines such as leptin and adiponectin play a role in the production of further inflammatory cytokines such as tumour necrosis factor-alpha (TNF-α) and interleukin (IL-) 6 from the adipose tissue^30,31^. Leptin further drives the T cell activation towards a Th1 phenotype with production of interferon γ^28^. These pro-inflammatory cytokines TNF, IL-6 and interferon γ in play a role in the inflammation of AD^28,32^. The adipose tissue is also the site for peripheral aromatization of androgens to oestrogen hormones such as oestrone and β-oestradiol^30^. The latter has been demonstrated to switch the initiating immune reaction from Th1 to Th2 type and also increase production of IL-4 and IL-13, characteristic of AD^27^. In addition to these inflammatory responses, obese individuals are observed to have an impaired epidermal barrier function as evidenced by an increased trans-epidermal water loss^28^.

The finding in our MR study that obesity has a causal relationship with AD is of great clinical importance as obesity could therefore be a modifiable risk factor for AD. Obesity itself has significant morbidity and mortality risks and therefore physicians should be cognizant of concomitant obesity and its related complications among AD patients. Although the importance of weight loss is widely appreciated by cardiovascular and metabolic medicine physicians, there is currently little appreciation of the importance of healthy weight in the management of dermatological conditions. Our results therefore have the potential to change current management guidelines for example, incorporating advisory regarding weight control through lifestyle modifications as a potential measure to alleviate AD.

Strengths of our study include the use of the two largest GWAS datasets for AD and obesity consisting of participants with European descent to date, with a total sample size of about 700,000 individuals and 21,000 AD cases and 95,000 controls respectively^14,15^. The genetic variants identified represent strong genetic instruments for evaluating causality. The correlation between genetic risk score based on the 941 SNPs and the BMI measurement in an independent validation cohort were 0.22^14^. The variance explained in this cohort from the SNPs were 6.0%. The AD GWAS dataset explain about 12.3% and 2.6% of variance for the previously established and newly identified SNPs^15^. This provided evidence that the MR assumption of relevance was valid in our study^33^.

We also assessed and supported the validity of the remaining two key MR assumptions of independence and exclusion restriction^33^. Modified IVW MR analysis, excluding potential pleiotropic factors based on published associations yielded effect estimates similar to our main analysis. Our MR-Egger intercept analysis also showed no evidence of confounding by horizontal pleiotropy^19^.

Various other MR methods (maximum likelihood, MR-Egger and weighted median method), in addition to the IVW method, were also performed as part of sensitivity analysis^17^. Results were consistent and similar in its direction and magnitude. Estimates from the MR-Egger and weighted median methods were not statistically significant and tend to have less precise confidence intervals.

Limitations include the use of GWAS datasets of participants of only European ancestry, which limits potential generalization to other populations such as Asians. Meta-analysis of studies from Asia has reported a significant relationship between AD and obesity^7^. However, as GWAS datasets from Asian participants have a much smaller sample size (weak instruments), they were not utilised in our current analyses^34,35^.

## Conclusions

In this study, taking a MR approach, we provide evidence to suggest that a higher BMI causally increases the risk of AD in individuals of European descent. There was no evidence that the reverse direction is true. Obesity can therefore act as a modifiable risk factor for AD, potentially changing our current clinical dermatological practice for management of patients with AD who are obese. In addition, our results open up new avenues to better understand the mechanistic pathways of BMI driving the risk of AD. This could in turn lead to the development of novel therapeutics to maintain skin health and prevent AD.

## Methods

Two-sample MR analysis with multiple genetic variants as instrumental variables using summarized data was performed in our study to assess the causal relationship and its strength between BMI and AD^36^. The MR analysis was performed in both directions to establish the direction of causality between BMI and AD. The validity of the instrumental variables are important for the MR analysis and are defined by three key assumptions: (1) genetic variants are associated with the exposure factor of interest (Relevance assumption); (2) genetic variants are independently associated with the outcome with no unmeasured confounders (Independence assumption); (3) genetic variants only affect the outcome through their effect on the exposure factors with no evidence of other horizontal pleiotropic factors (Exclusion restriction assumption)^33^. Data was extracted from the two largest GWAS reported to date on BMI and AD to fulfil the relevance assumption of MR. We carried out sensitivity analyses by excluding SNPs that have associations with possible confounders in the MR analyses, and performed an MR-Egger test of pleiotropy, to assess the validity of the independence and exclusion restriction assumptions respectively.

### GWAS of BMI and AD

The following GWAS for BMI and AD provided genetic risk variants information for the MR analysis. Genetic risk variants for BMI were determined using the combined analysis of the Genetic Investigation of ANthropometric Traits (GIANT) consortium and the UK biobank dataset of about 700,000 individuals with European ancestry in total^14^. The GIANT consortium is a joint GWAS and metabochip meta-analysis of 114 studies that measured BMI as its phenotype of interest. Details of included studies (assessment and definitions of phenotypes, genotyping and quality control process) in the BMI GWAS analysis and UK biobank are provided in Supplementary Table S4. Meta-analysis of summary statistics from these two studies identified 941 near-independent single nucleotide polymorphisms (SNPs) associated with BMI at a revised genome-wide significance threshold of P < 1 × 10^−8^). These SNPs were identified using an approximate conditional and joint multiple-SNP (COJO) analysis that takes into account LD (linkage disequilibrium) between SNPs at a given locus. As part of sensitivity analyses, we did a between-SNPs LD r^2^ analysis and excluded SNPs across a range of r^2^ thresholds (> 0.1, > 0.2, > 0.5) followed by distance pruning at < 500 kb as well as SNPs with r^2^ thresholds (r^2^ > 0.001 and > 0.01) within 1000 kb apart (Supplementary Table S2a).

Similarly, genetic risk variants for atopic dermatitis were determined using the dataset of the EArly Genetics and Life course Epidemiology (EAGLE) Consortium^15^ of 21,000 cases and 95,000 controls. This GWAS meta-analysis analyzed AD case–control status in 22 individual cohorts of European ancestry. Details of included studies in the AD GWAS analysis are provided in Supplementary Table S5. The study reported 24 SNPs reaching genome wide significance (*P* < 5 × 10^−8^) for AD risk. These SNPs were independent with at least 4 Mb apart.

### Statistical analysis

The two-sample MR strategy was adopted in our analysis^37^. The SNP-exposure effects and the SNP-outcome effects were obtained from separate GWAS datasets. First, summary level SNPs-exposure associations were extracted from the first GWAS dataset on exposure as MR instruments. These instruments SNPs-outcome associations were then extracted with the second GWAS dataset on outcome. Any missing exposure associated variants in the outcome GWAS dataset were replaced by linkage disequilibrium (LD) proxies of a minimum of 0.6. Only one SNP out of the 941 SNPs (rs11172702) in the BMI GWAS dataset had missing SNP-outcome effect measure in the outcome (AD) dataset that required a proxy SNP (rs11172644; LD: r^2^ = 1) for analysis. Comparatively, 10 out of the 24 SNPs (rs10199605, rs12730935, rs2227483, rs2592555, rs4809219, rs10791824, rs12188917, rs2212434, rs2918307 and rs6419573) had missing SNP-outcome effect measures in the BMI dataset and required proxy SNPs of LD r^2^ values between 0.67 to 1.00. Four missing SNPs (rs112111458, rs4713555, rs145809981 and rs61813875) had no proxy SNPs with adequate LD r^2^ values of greater than 0.6 and were therefore excluded from the analysis.

The exposure and outcome effects were then harmonized. This involved identification of SNP variants of unmatched effect and alternate alleles between the two datasets and correction by switching the direction of their effect estimates and corresponding effect allele frequencies. We searched for palindromic SNPs that might have inverted the direction of effect in the BMI and AD GWAS datasets, and found no evidence for such inversion. There was therefore no need to realign any of the palindromic SNPs used. Individual SNP estimates were first calculated using the Wald ratio method. The Wald ratio refers to the ratio estimate of the effect of the variant-outcome divided on the effect of variant-exposure. The standard error of the ratio estimate was approximated using the delta method^38^.

### Inverse variance weighted MR

An MR estimate using multiple SNPs was obtained by performing a random effects inverse variance weighted (IVW) meta-analysis of each Wald ratio of corresponding SNP^18^. The IVW method assumes that all SNPs are valid instruments or any underlying horizontal pleiotropy is balanced across the SNPs^39^. In order to examine the robustness of the estimates, we also estimated the causal effect estimate of exposure on outcome using three other MR methods: maximum likelihood method, weighted median-based method and MR-Egger regression analysis.

### Maximum likelihood method

The causal effect parameter was estimated from a model that assumed a linear relationship between exposure and outcome and a bivariate normal distribution for the genetic variants^40^. Standard errors for the maximum-likelihood estimates were obtained using the inverse Hessian matrix. This method made similar assumptions as the IVW approach but provides more reliable estimates in the presence of measurement error in SNP-exposure effects^41^.

### Weighted median-based method

This approach examines the median effect of all available SNPs and only requires half the SNPs to be valid instruments for the effect estimate to be unbiased. 50% of the weights in the analysis will be from ratio estimates smaller or equal to the weighted median^42^.

### MR-Egger regression analysis

The MR-Egger regression analysis is an adaptation of the IVW analysis by allowing a non-zero intercept that is estimated as part of the analysis^43^. This relaxed the assumption of no horizontal pleiotropy and allowed net horizontal pleiotropic effect across all SNPS to be unbalanced, or directional. However, any horizontal pleiotropic effect should not correlate with SNP-exposure effects. MR-Egger intercept test was also performed as part of the MR-Egger regression analysis to assess whether the genetic variants have directional horizontal pleiotropic effects on the outcome. It is a test of whether the intercept of the MR-Egger regression analysis significantly differs from zero.

### Power calculation of MR analysis

Calculations for statistical power for our MR analyses were performed according to Brion et al. using their web-based application^44^. Power calculations of the MR analysis of BMI upon AD were provided for a range of true odds ratio of AD per unit increase of BMI (Supplementary Fig. S2)^44^. Calculations were based on a sample size of 116,000 (21,000 AD cases and 95,000 controls) in the outcome dataset (i.e. AD GWAS study) and a type 1 error rate of 0.05. Proportion of variance explained for the association of the included SNPs (n = 941) with BMI (exposure variable) was 0.06. Our study had 80% power to detect an odds ratio for AD of 1.09 per unit (kg/m^2^) increase of BMI in the MR analysis.

Power calculations of the MR analysis of AD upon BMI were provided according to a range of values for the true underlying causal association between AD and BMI (Supplementary Fig. S2)^44^. These was based on a sample size of 681,275 in the outcome dataset (i.e. BMI GWAS study) and a regression coefficient of 0.10 kg/m^2^ for the observational association between AD and BMI according to a recent cohort study by Shalom et al.^45^ The variances for AD (exposure) and BMI (outcome) used were 0.148 and 16 kg^2^/m^4^ respectively based on AD and BMI GWAS datasets^14,15^. Proportion of variance explained for the association of the included SNPs (n = 20) with AD (exposure variable) was 0.026. Our study had 80% power to detect a 0.220 kg/m^2^ increase in BMI arising from the presence of AD at a type 1 error rate of 0.05.

### Sensitivity analysis

In order to improve the reliability of our MR results, we performed sensitivity analyses by excluding potential pleiotropic variants in our MR analyses. The associations of the genetic variants used as genetic instruments with potential confounders of BMI and AD were annotated using the PhenoScanner V2 database^46,47^. (Supplementary Tables S6, S7). Potential confounders considered for the BMI SNPs included education, behavioural factors such as tobacco smoking, alcohol ingestion and physical activity, psychiatric diseases and psychological well-being (anxiety, depression)^48–52^. Potential confounders considered for the AD SNPs included education, alcohol ingestion psychiatric diseases and psychological well-being (anxiety, depression)^48–52^.

Given that there might be a certain degree of measurement error in the SNP exposure associations, resulting in departure of the no measurement error (NOME) assumption, we also estimated the attenuation of effect estimates in IVW and MR-Egger methods using F statistics and I^2^ respectively. Both range from 0 to 100%, with values close to 100% suggestive of minimal attenuation^53,54^.

The attenuation of the effect size as a result of departure of NOME assumption in the IVW method can be estimated by measuring the instrument strength (*F* statistics) for the genetic variant used^53^. *F* statistic for each variant is calculated as the ratio of its effect size estimate to the variance of its SNP-exposure association, a weighted *F* statistic is then measured across all the SNPs used in the MR experiment. Degree of attenuation of effect estimates resulting from departure of NOME assumption is then estimated by:

$$\frac{{\stackrel{-}{F}}_{GX}-1}{{\stackrel{-}{F}}_{GX}}$$

In contrast, MR Egger estimates are not governed by *F* statistics and are better assessed using I^2^_GX_ estimate^54^. I^2^_GX_ represents the true variance of SNP-exposure associations divided by the variance of the SNP-exposure estimates.

TwoSampleMR (version 0.4.26) and Mendelian Randomization (version 0.4.1) packages in R statistical software (RStudio version 1.2.1335) were used to perform data clumping of SNPs and two-sample Mendelian randomization analysis respectively. The MR experiment is summarized in Fig. 1.

## Supplementary information

Supplementary Figures.

Supplementary Tables.

## Data Availability

All data generated or analysed during this study are included in this published article and its Supplementary Information files.
