# Supplementary material for: Investigating causal relationships between Body Mass Index and risk of atopic dermatitis: a Mendelian randomization analysis
Source: Sci Rep. 2020 Sep 17;10:15279. doi: 10.1038/s41598-020-72301-2 (PMC7498603; doi:10.1038/s41598-020-72301-2)
Supplement: Supplementary file 1 — Supplementary Figures. [file 41598_2020_72301_MOESM1_ESM.pdf]

Title: Investigating causal relationships between body mass index and risk of atopic dermatitis: a Mendelian randomization analysis

Authors: Yik Weng Yew[1,2], Marie Loh[2,3], Steven Tien Guan Thng[1,4], John C Chambers[2,3]

Affiliations:

[1] National Skin Centre, Singapore

[2]Lee Kong Chian School of Medicine, NTU, Singapore

[3]Imperial College London, UK

[4]Skin Research Institute Singapore

Supplementary Figures Legends

Supplementary Figure 1a: Schematic diagram of MR analysis of BMI upon atopic dermatitis (AD). Significant ($P < 1 \times 10{-}8$) and independent BMI SNPs (based on an approximate conditional and joint multiple-SNP (COJO) analysis that takes into account LD (linkage disequilibrium) between SNPs at a given locus) from BMI GWAS were used as instrumental variables to assess the causal effect of BMI upon AD.

Supplementary Figure 1b: Schematic diagram of MR analysis of atopic dermatitis (AD) upon BMI. Significant ($P < 5 \times 10^{-8}$) and independent AD SNPs (at least 4 MB(mega base pairs) apart) from AD GWAS were used as instrumental variables to assess the causal effect of AD upon BMI.

Supplementary Figure 2. Power curves with a binary variable outcome (Atopic Dermatitis, AD) and continuous outcome (Body Mass Index, BMI). Left panel: For different values of true odds ratio (OR) of AD per unit (kg/m2) increase in BMI, the dashed line shows 80% power to detect OR of 1.09 per unit increase of BMI. This was based on a sample size of 116,000 (21,000 AD cases and 95,000 controls) in the outcome dataset (i.e. AD GWAS), a type 1 error rate of 0.05 and proportion of variance explained for the association of the included SNPs (n=941) with BMI (exposure) 0.06. Right panel: For different values of the true causal effect (β) between AD and BMI, the dashed line shows 80% power to detect a 0.220kg/m2 increase in BMI arising from the presence of AD. This was based on a sample size of 681,275 in the outcome dataset (i.e BMI GWAS), a type 1 error rate of 0.05 and proportion of variance explained for the association of the included SNPs (n=20) with AD (exposure) = 0.026.

Supplementary Table Legends

Supplementary Table 1: List of SNPs for body mass index (BMI) and their effect on risk of atopic dermatitis (AD).

Supplementary Table 2a: List of SNPs removed at different r2 threshold and within 500kb/1000kb apart.

Supplementary Table 2b: Sensitivity Analyses excluding body mass index (BMI) SNPs with different r2 thresholds and within 500kb/1000kb apart.

Supplementary Table 3. List of SNPs for atopic dermatitis (AD) and their effect on body mass index (BMI).

Supplementary Table 4. Study design, number of individuals, sample quality control, genotyping methods, and quality control of SNPs for genome-wide association studies and metabochip studies (BMI).

Supplementary Table 5. Study design, number of individuals, sample quality control, genotyping methods, and quality control of SNPs for genome-wide association studies (atopic dermatitis).

Supplementary Table 6: Body Mass Index (BMI) SNPs and annotated associations with possible confounders.

Supplementary Table 7: Atopic Dermatitis (AD) SNPs and annotated associations with possible confounders.

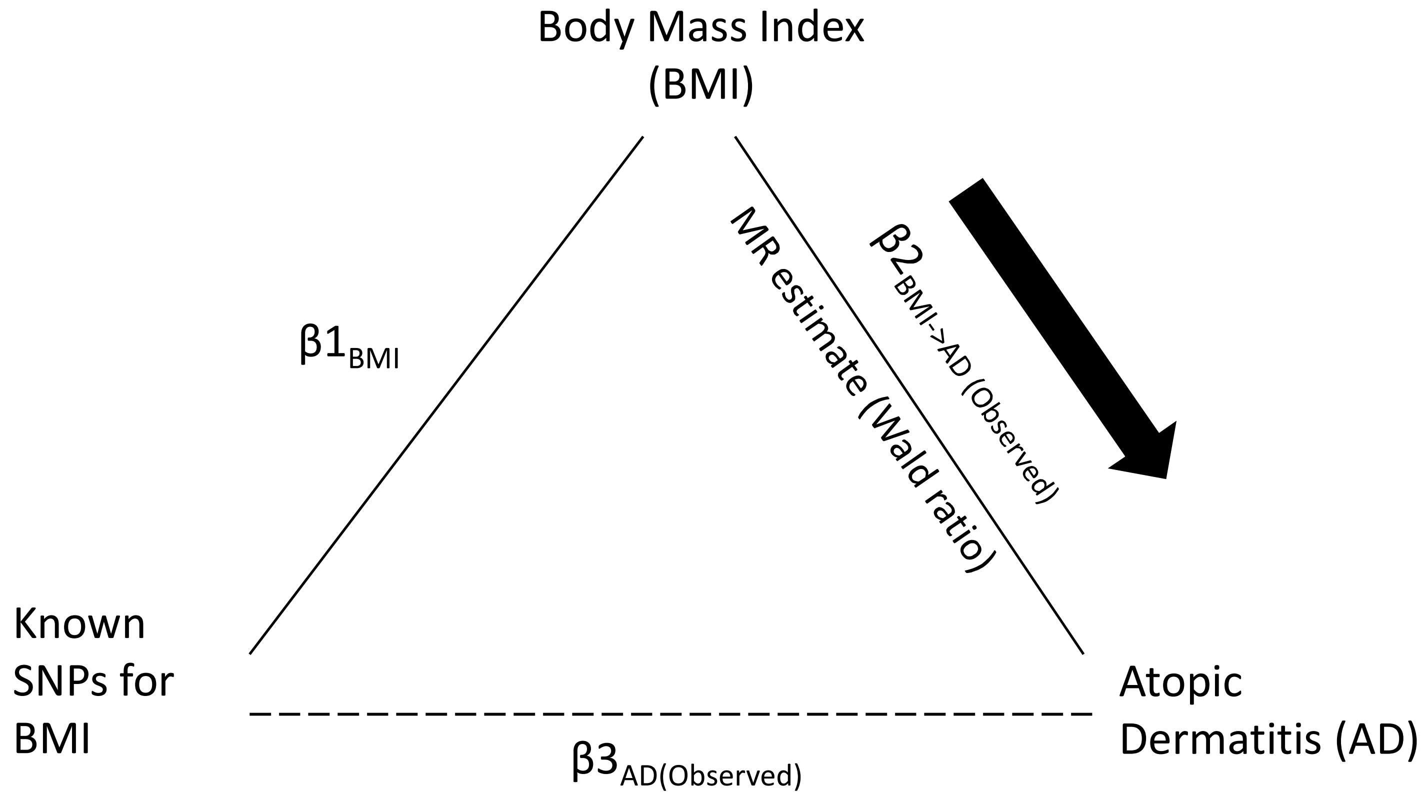

Supplementary Figure 1a. Schematic diagram of MR analysis of BMI upon atopic dermatitis (AD). Significant ($P < 1 \times 10-8$) and near-independent BMI SNPs (based on an approximate conditional and joint multiple-SNP (COJO) analysis that takes into account LD (linkage disequilibrium) between SNPs at a given locus) from BMI GWAS were used as instrumental variables to assess the causal effect of BMI upon AD.

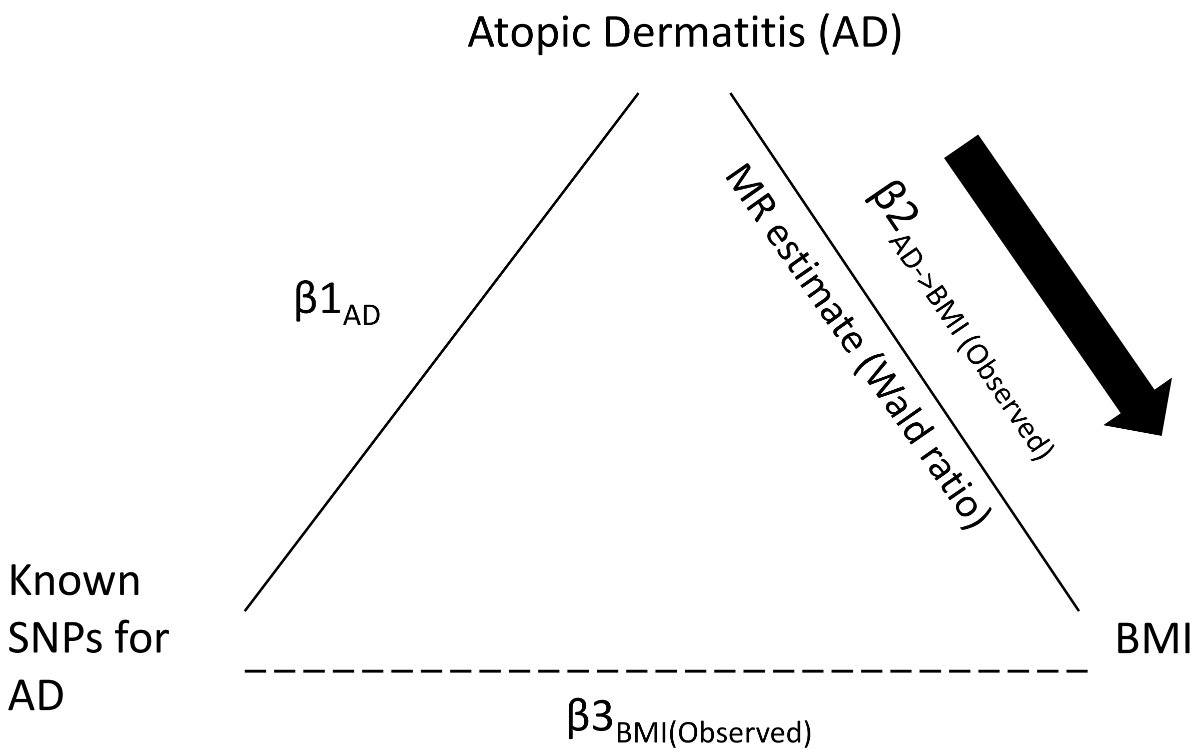

Supplementary Figure 1b. Schematic diagram of MR analysis of atopic dermatitis (AD) upon BMI. Significant ($P < 5 \times 10{-}8$) and independent AD SNPs (at least 4 MB(mega base pairs) apart) from AD GWAS were used as instrumental variables to assess the causal effect of AD upon BMI.

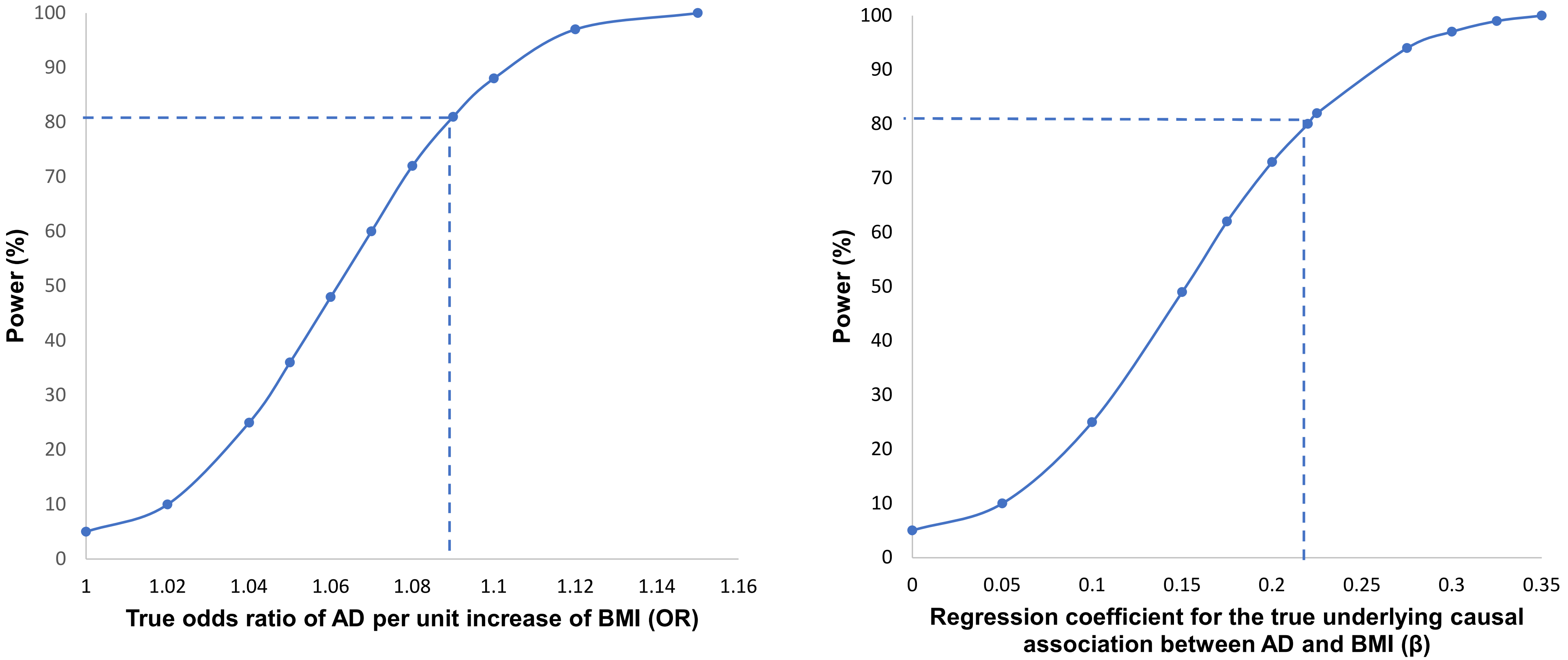

Supplementary Figure 2. Power curves with a binary variable outcome (Atopic Dermatitis, AD) and continuous outcome (Body Mass Index, BMI). Left panel: For different values of true odds ratio (OR) of AD per unit (kg/m^2) increase in BMI, the dashed line shows 80% power to detect OR of 1.09 per unit increase of BMI. This was based on a sample size of 116,000 (21,000 AD cases and 95,000 controls) in the outcome dataset (i.e. AD GWAS), a type 1 error rate of 0.05 and proportion of variance explained for the association of the included SNPs (n=941) with BMI (exposure) = 0.06. Right panel: For different values of the true causal effect (β) between AD and BMI, the dashed line shows 80% power to detect a 0.220kg/m^2 increase in BMI arising from the presence of AD. This was based on a sample size of 681,275 in the outcome dataset (i.e BMI GWAS), a type 1 error rate of 0.05 and proportion of variance explained for the association of the included SNPs (n=20) with AD (exposure) = 0.026.
